# Can conditional cash transfers improve maternal health care? Evidence from El Salvador's *Comunidades Solidarias Rurales* program

**DOI:** 10.1002/hec.4012

**Published:** 2020-03-02

**Authors:** Alan de Brauw, Amber Peterman

**Affiliations:** ^1^ Markets, Trade and Institutions International Food Policy Research Institute Washington DC United States; ^2^ Social and Economic Policy UNICEF Office of Research–Innocenti Florence Italy

**Keywords:** conditional cash transfers, maternal health, El Salvador, regression discontinuity design, implicit partition

## Abstract

There is growing evidence on positive human capital impacts of large, poverty‐focused cash transfer programs. However, evidence is inconclusive on whether cash transfer programs affect maternal health outcomes, and if so, through which pathways. We use a regression discontinuity design with an implicit threshold to evaluate the impact of *Comunidades Solidarias Rurales* in El Salvador on four maternal health service utilization outcomes: (a) prenatal care; (b) skilled attendance at birth; (c) birth in health facilities; and (d) postnatal care. We find robust impacts on outcomes at the time of birth but not on prenatal and postnatal care. In addition to income effects, supply‐side health service improvements and gains in women's agency may have played a role in realizing these gains. With growing inequalities in maternal health outcomes globally, results contribute to an understanding of how financial incentives can address health systems and financial barriers that prevent poor women from seeking and receiving care at critical periods for both maternal and infant health.

## 1 INTRODUCTION

Large‐scale cash transfer programs have become a mainstay in social protection and poverty reduction strategies in low‐ and middle‐income countries globally, reaching over 780 million beneficiaries (Honorati et al., 2015). Although specific design components vary, most government‐run programs in Latin America and the Caribbean (LAC) condition transfers, modeled after México's *Prospera* (formerly *Progresa* and *Oportunidades*). The typical national conditional cash transfer (CCT) in LAC requires beneficiaries to comply with co‐responsibilities related to child health and/or education to remain eligible for transfers. Beneficiaries may also be required to attend periodic trainings focused on health and nutrition information and behavior change. A large literature explores the impact of cash transfers on children's education, health, nutrition, and household‐level poverty‐related outcomes (e.g. Baird et al., 2013; Glassman et al., 2013; de Groot et al., 2017; Hidrobo et al., 2018).

As cash transfer programs continue to expand, there is growing interest in examining maternal health impacts, defined as the health of women during pregnancy, childbirth, and postpartum periods. In a desk review of CCTs and health, Morris (2010) concludes that “in spite of the remarkable success of CCT programs in changing household behaviors, it is most unlikely that they have contributed anything to the global effort to reduce child and maternal mortality.” He argues that in terms of health, the “greatest failure” of CCTs in LAC is the “neglect of the very period in which the need for behavior modification is greatest: labor, delivery and the immediate postpartum recovery phase (p. 229).” This claim is partially driven by a lack of evidence, as detailed data on maternal and reproductive health has either not been collected or not analyzed within CCT evaluations. Although more recent cash and voucher programs are explicitly designed to improve maternal health, the targeting of maternal health outcomes has been limited. Thus, the most current evidence comes from technical reports examining maternal health as a secondary outcome.

Despite broader objectives of poverty alleviation and increasing human capital, large‐scale CCTs are well positioned to influence maternal health outcomes. Such influence could occur through six distinct pathways: (a) through an income effect, particularly if it is (b) coupled with shifts in intrahousehold power or autonomy favoring women; (c) lower prices, attained by removing or reducing health service user fees, lowering the price; (d) through explicit or implicit program conditionalities related to maternal health utilization; (e) by providing information causing beneficiaries to update their beliefs about the value of maternal health care; and (f) supply‐side improvements in health facilities.

When cash transfers are directed to women, household income and the share of it controlled by women increases. Consequently, one might expect increased demand for or utilization of health services, specifically related to women's health. Intrahousehold shifts in demand may also occur from additional program components, which increase women's mobility (e.g. Ambler & de Brauw, 2017) or the size of women's social networks. Third, incentives for health care utilization improve if CCTs remove additional demand‐side barriers, by reducing or eliminating fees for services such as prenatal care or care at the time of delivery. Further, programs may include prenatal or postnatal visits as part of household co‐responsibilities, effectively contracting women to meet health service utilization requirements to maintain eligibility. Programs may also stimulate demand for care through health or nutrition trainings, often targeted towards women and offered in parallel with transfer payments. Finally, CCTs may increase the quality and quantity of health services through investment in infrastructure and supply‐side improvements of clinics in treatment communities. The likelihood of distinct pathways for each case will depend, upon service availability and improvement, the local context and norms surrounding maternal health care, and/or on transfer design features (e.g. transfer size, duration, conditionalities, size, and targeting within households).

This paper contributes to the literature on CCT impacts on maternal health service utilization, focusing on the period before, at and after childbirth. We use a regression discontinuity design (RDD) and a unique implicit threshold to evaluate the impact of El Salvador's national CCT program *Comunidades Solidarias Rurales* (CSR) on the following outcomes: (a) adequate prenatal care, (b) skilled attendance at birth, (c) birth in health facilities and (d) the receipt of postnatal care. 1The program name became *Comunidades Solidarias Rurales* in 2009 after a change in government, so we refer to the program throughout as CSR, although the program was called *Red Solidaria* during the evaluation.Like many other CCTs in LAC, CSR consists of a bimonthly transfer given to mothers of school age children and children under five, conditional on school enrollment and attendance and health clinic visits, respectively. In addition, pregnant women were required to attend prenatal visits and all beneficiaries were invited to participate in monthly trainings related to health and nutrition knowledge and behavior change. The data used in analysis were collected in two rounds in early and late 2008, by the International Food Policy Research Institute (IFPRI) in collaboration with the *Fundación Salvadoreña para El Desarrollo Económico y Social* (FUSADES) and the Government of El Salvador.

To identify the impacts of CSR on maternal health service utilization, we use RDD combined with a double difference estimator. We exploit the sequential timing of program entry, using a group entering CSR in 2006 as the treatment and a group entering in 2007 as the control group. We find strong and robust impacts of CSR on skilled attendance at birth and on birth in a hospital setting, but no impacts on the receipt of adequate prenatal care, or the receipt of any postnatal care. These results are robust to a number of sensitivity analyses including varying the RDD bandwidth, kernel construction, adding control variables, and alternative constructions of variables measuring outcomes. Investigation of impact pathways indicate that beyond income effects associated with CSR transfers, supply‐side health service improvements and gains in women's decision‐making agency are likely to have been important factors.

The paper proceeds as follows. In the next section, we provide a critical review of the existing evidence on CCTs and maternal health, focusing on large‐scale programming and on service utilization outcomes. Third, we discuss details about the implementation of CSR relevant to the paper, and fourth, we describe the data that will be used in the analysis. The fifth section describes our RDD strategy in more detail. The sixth section presents and discusses results, and the final section concludes with discussion and policy implications.

## 2 CCTS AND MATERNAL HEALTH: CURRENT KNOWLEDGE

A systematic review conducted in 2013 linking CCTs to maternal and newborn health outcomes included both broad poverty‐target programs and narrowly focused maternal health voucher programs (Glassman et al., 2013). It concluded that CCTs have been relatively successful at reducing barriers to service utilization including prenatal care, skilled attendance and facility births, and outcomes such as tetanus toxoid vaccination for mothers and low birth weight. However, the authors found neither measurable impacts on fertility (a positive finding) nor maternal or newborn mortality. Despite promising results related to service utilization, causal pathways are not well defined, so it is not clear what design components are responsible for impacts across studies. To provide additional detail, we focus on poverty and human‐capital targeted cash transfers in the remaining discussion. 2Targeted voucher schemes typically involve a one‐time incentive to induce women to give birth in facilities and have been primarily evaluated in South Asia (e.g. Lim et al., 2010; Powell‐Jackson & Hanson, 2012; Nguyen et al., 2012). The behavioral and poverty pathways to impacts from vouchers are likely to be substantially different from CCTs.


Maternal health outcomes have been most documented for México's *Progresa* (now *Prospera*). Urquieta‐Salomon et al.( 2009) find the program had no impact on skilled attendance at birth among rural women, except among a select group of high fertility women who had one birth just before and one just after program initiation. In more recent data, Sosa‐Rubi et al. (2011) find that younger cohorts (aged 15 to 24 years) have a substantially greater likelihood of choosing a physician or nurse than a traditional midwife. Barber and Gertler (2009) use a quality of care index and find beneficiaries received 12.2% more procedures than nonbeneficiaries during prenatal visits, attributing the result to the notion that women become more active and informed health consumers through CCT participation. Finally, Barber (2009) finds the program increased the rate of caesarean section at birth by 5.1 percentage points overall, 7.5 percentage points among women exposed to the program for over 5 months and particularly among women giving birth in government‐run facilities. Therefore, in relation to the main health utilization outcomes of interest, there is some promising evidence that the longest‐running national program in LAC has resulted in changes in skilled attendance at birth for younger women, as well as quality of prenatal care.

Other mixed evidence from CCTs in LAC comes from Brazil, Honduras, Guatemala, and Uruguay. In Brazil, the *Bolsa Alimentição* program did not have significant impacts on either timing of the first prenatal visit or the total number of visits (IFPRI, 2003). However, the sample size was small (287 women), so statistical power to detect impacts was lacking. In Honduras, a 2‐year CCT pilot found an 18 to 19 percentage point increase in the receipt of five or more prenatal care visits, with no associated impacts on postnatal care visits. However, prenatal care outcomes were not balanced at baseline, and government facility data did not confirm this finding, calling it into question (Morris et al., 2004). More promising evidence comes from Guatamala's CCT *Mi Familia Progresa*, where positive impacts were found on skilled attendance and prenatal care, particularly among indigenous women (Gutierrez, 2011). Finally, Amarante et al. (2016) use admnistrative data and find that Uruguay's *Plan de Atencion National a la Emergencia Social* (an emergency unconditional cash transfer) reduced low birthweight, which they hypothesize is due in part to mothers improved nutrition during pregnancy. 3Although in theory the program was designed as conditional on health visits for and school attendance for children, as well as health visits for pregnant women, conditions were not formally stipulated until late in the program and in practice were never enforced. Therefore, although some beneficiaries may have perceived such conditions resulting in behavioral changes, the authors analyze and frame the program as a UCT.There were no impacts on the number of prenatal visits, but there was a simultaneous increase in births in public facilities (3.1 percentage points) and a decrease in births assisted by a medical doctor (2.8 percentage points). In sum, whereas there is growing evidence that CCTs in LAC have positive impacts on maternal health service utilization, the literature is relatively inconclusive and lacks detail on impact pathways. Furthermore, studies often examine a single outcome rather than looking at a combination of services, service quality, or health outcomes.

## 3 EL SALVADOR AND *COMUNIDADES SOLIDARIAS RURALES*

El Salvador began implementing CSR in 2005 as a pilot, and it was fully implemented by 2009, benefitting over 75,000 households at its peak in 2013 (Honorati et al., 2015). CSR is geographically targeted through a two‐step process. First, the municipalities in El Salvador were all grouped by levels of extreme poverty using a procedure called partitioned cluster analysis based on two indicators: the poverty rate, measured using data collected at the municipality level from 2001 to 2004, and the prevalence of severe stunting (the proportion of children under three standard deviations below the mean height‐for‐age z‐score) among first graders in the 2000 height census. The 100 municipalities in the two highest poverty groups, which we call the “High Poverty” and “Moderate Poverty” groups, were targeted for the program. 4These two groups of municipalities were called *pobreza extrema severa* and *pobreza extrema alta* by the government.The program was then rolled out between 2005 and 2009, beginning with high poverty municipalities in 2005 and 2006, followed by moderate poverty municipalities between 2007 and 2009.

When the CSR program was being established, indicators related to maternal health in El Salvador suggested room for improvement. FESAL (2009) reported about 78% of women of reproductive age nationwide completed the five recommended prenatal visits, 84% gave birth in hospitals, and 54% completed postnatal visits in the 6 months after giving birth. These figures were all increases from previous estimates (2002–03) but varied substantially by region and between rural and urban areas, suggesting they are lower among the rural poor.

To target households within municipalities, the implementing agency, *Fondo de Inversión Social para el Desarrollo Local* (FISDL), first carried out a municipality census on a rolling basis to determine program eligibility. The census took place during the same year as the muncipality entered CSR. Households were eligible for the health transfer if either a member was pregnant at the time of the census or if a child residing in the household was under 5 years of age. Households were eligible for the education transfer if any children aged 6 to 15 residing in the household had not completed primary school. The health transfer was conditioned on growth monitoring visits every 2 months for children, vaccination status, and prenatal care for pregnant women. Transfer amounts are $15 (USD) per month for households eligible for the health or education benefit alone, and $20 per month for households eligible for both health and education benefits, and do not vary by the number of children in the household. Monthly training sessions were offered at local village centers on topics ranging from education, nutrition, health and women's or child's rights. Although attendance was taken at training sessions, a transfer receipt is not conditioned on beneficiary attendance. 5However, the evaluation surveys indicate that 97% of beneficiaries believed transfers were conditional on meeting attendance. The government simultaneously implemented a set of infrastructural improvements, including water and sanitation projects and health systems investments. 6Health systems investments occurred through a simultaneous, but separate, program run by the Ministry of Health, called “Extension of the Coverage of Basic Services.”Such improvements took place in almost all participating municipalities.

In practice, transfers worked as follows. Eligible children and mothers went to a central location in the municipality once every 2 months on a designated day to collect the transfer. Their name and card were checked against a list of eligible beneficiaries based on meeting all conditions, and they would receive their transfer if so. If conditions were not being met, beneficiaries would first incur a “penalty,” in which they would receive a partial transfer, and if they continued failing to meet conditions, they would be dropped from the program. A local official was in charge of monitoring conditions and reporting them to the central FISDL office. In practice, according to qualitative and quantitative evaluation surveys, conditions were well understood and typically met.

## 4 DATA AND METHODOLOGY

The data used for this paper were collected by FUSADES in collaboration with IFPRI, and the sample and survey were designed explicitly to evaluate the impact of CSR on indicators of infant and maternal health, education, and nutritional status. The first survey round was collected in January and February, 2008, and a second survey was collected between late September and November, 2008. The survey form included sections on household demographics, education, health, time allocation, labor, housing and durables, agriculture, migration, expenditures, and community participation in programs, including CSR. The entire sample includes 100 *cantones*, two each, in 50 municipalities.

This paper uses a subset of the entire data set collected for the impact evaluation. First, it uses the subsample of 22 municipalities that entered CSR in either 2006 or 2007. Before the 2007 entry group begins receiving transfers, the 2006 entry group can be considered the treatment group and the 2007 group the control group. We therefore also filter out all births or pregnancies completed after the 2007 entry group began receiving payments, which took place no earlier than late July, but typically took place in late September.

**Figure 1:**
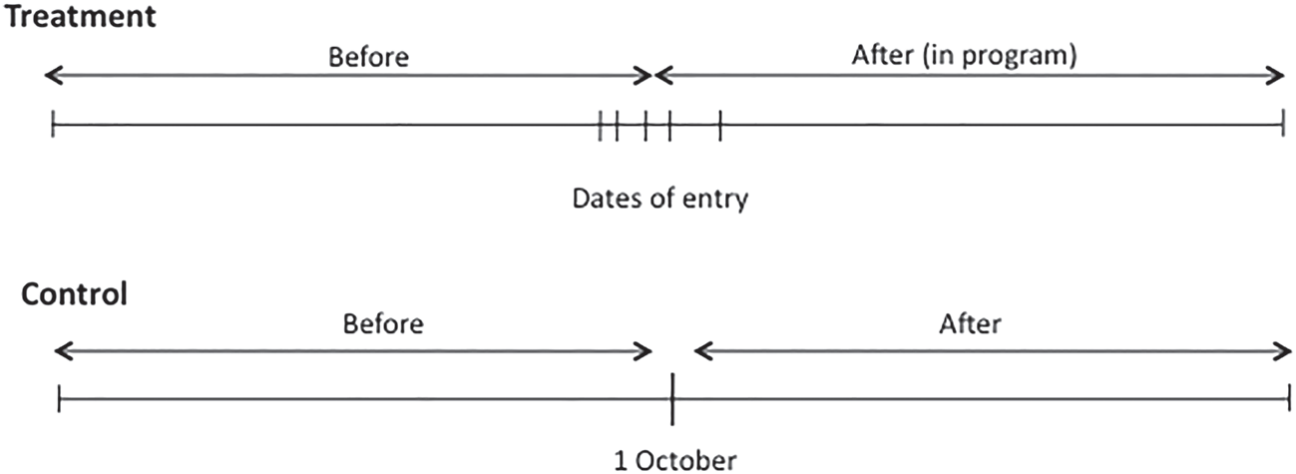
Illustration of “treatment” and “control groups” for maternal health outcomes

Data used for the impact evaluation include the following. First, we use fertility histories in both the 2006 and 2007 entry groups to isolate births or pregnancies that occurred both before and after payments began in 2006 for the treatment group. 7See Figure 1 for an illustration of this discussion.For the 2006 entry group, the date of the first payment in each municipality acts as a cut‐off between the before and after periods. For indicators measured at or after birth, we consider the birth as pre‐program if occurring before the first payment date, and as post‐program if occurring after the payment date. The payment date works as the cutoff between the before‐program and after‐program periods because health training sessions did not start until payments started, and the health‐seeking behaviors studied are not likely to occur until after the program starts. 8There is no reason to believe that behavior would change related to maternal health during the 2 month period between the municipal census and the first payment.For prenatal care indicators, we define the cutoff period slightly differently, as the woman must be at least 2 months pregnant by the time of initial payment as a cutoff. We use 2 months as the threshold, as women are typically aware of the pregnancy by then, and thus there is potential to change behavior, such as initiating a health clinic visit or prenatal care. If women did not report being pregnant at the time of the census, then they are not eligible for transfers associated with CSR. For the 2007 entry group, we use October 1, 2006, as the cutoff for before and after, as it is the midpoint of dates at which the first payment took place in the 2006 entry group. These definitions allow us to estimate impacts in a double difference framework.

The sampling strategy for data collection was explicitly designed to ensure adequate sample sizes to examine maternal and young child health outcomes. The initial survey used municipality‐level census data as a sampling frame, and within each *cantón*, 15 households with children under 3 years old or with a pregnant woman resident at the time of the census were included in the sample. 9The sample also included 15 households with children between the ages of 6 and 12 selected randomly within each *cantón* from census lists, for a total of 30 households per *cantón*.Households were tracked in the second survey if the household remained in each specific demographic group (children under 3 or children aged 6 to 12 years at present). To replace households no longer in those demographic groups, the sample was replenished either from the baseline census or from a list of recent births collected at health clinics. All additions to the sample for this paper, however, either come from the panel households or the replenishment sample, as the births between early and late 2008 occur after both treatment and control groups are receiving transfers.

A module in both the first and second surveys specifically focused on maternal health and collected pregnancy histories for all women and adolescent girls over the age of 12. In the first survey, women were asked about all current and previous pregnancies occurring since the beginning of 2006. In the second survey, households were asked only about pregnancies that had occurred in the past 12 months. Among mothers that lived in households interviewed in both rounds, we carefully examined the combined data on pregnancies to ensure that each pregnancy was included only once. The resulting sample includes 548 women with valid responses for attendance at birth and birth in facility, and 510 women with valid responses for prenatal and postnatal care.

### 4.1 Outcome indicators

We examine four main outcome indicators reflecting different stages of health care utilization over the pregnancy and birth periods: (a) adequate prenatal care, (b) skilled attendance at birth, (c) birth in health facilities, and (d) postnatal care. Adequate prenatal care is defined as at least five visits over the pregnancy period as recommended by the Salvadoran Ministry of Health. Skilled attendance at birth is defined as attendance by general practitioner doctors, obstetricians/gynecologists, and nurses, again as recommended by government guidelines. Birth in facilities is defined as birth in a government or private hospital and excludes births reported at health centers or at mobile health clinics. Postnatal care attendance is defined as meeting with a health professional within 2 weeks of giving birth for a check‐up. 10We also tested a version of this variable in which we extend the time frame for a postnatal visit to 6 weeks; results do not change.


### 4.2 Statistical identification

**Figure 2:**
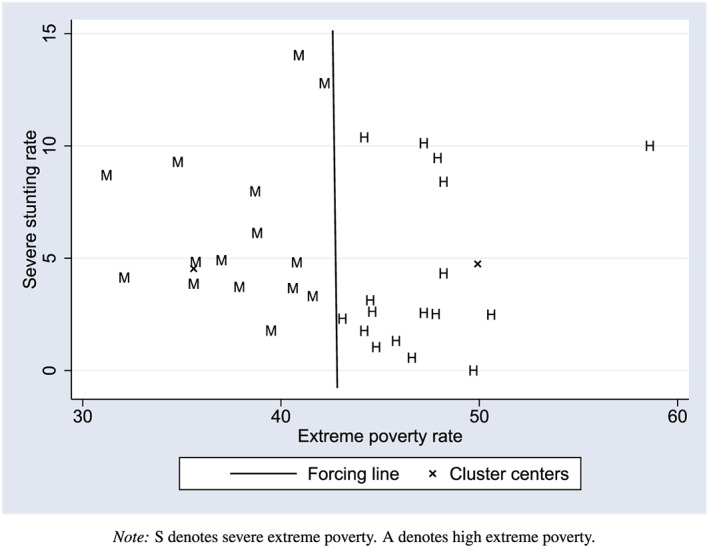
Illustration of boundary between severe and high extreme poverty groups for all municipalities entering *Comunidades Solidarias Rurales* in 2006 and 2007 [Colour figure can be viewed at wileyonlinelibrary.com]

To identify the impacts of CSR, we need to define comparable treatment and control groups. We use the process that determined eligibility for CSR to do so. Eligibility was defined by the Salvadoran government using a procedure called partitioned cluster analysis, leading to the high and moderate poverty groups. 11Appendix S1 discusses partitioned cluster analysis and the implicit partition used as a regression discontinuity design threshold in more detail. For further information about the boundary definition and the estimator, see de Brauw and Gilligan (2011).The two variables used for partitioning were the municipality level poverty rate and the severe stunting rate among first graders in a census conducted in 2000 (Figure 2). Important for statistical identification is that there is an implicit partition between the two groups—any municipality is either in one group or another, as membership for each grouping is defined by proximity to a cluster center. It is possible to therefore completely separate the 2006 and 2007 entry groups as they are part of the high and moderate poverty groups, respectively. As a result, RDD can be used to identify impacts. We call the line that separates the two groups the cluster threshold, and then, the distance from each municipality to that line is called the distance to cluster threshold and is used as the RDD forcing variable. 12In RDD, the variable for which a change in status occurs is called the forcing variable. The value of the forcing variable for which the change in status occurs is called the threshold. Here, the change in status is from the high poverty to the moderate poverty group.Intuitively, the municipalities closer to the implicit threshold should be quite comparable.

### 4.3 Identification of Impacts of CSR using RDD

Following Lee and Lemieux (2010), a local linear regression can be written as a parametric linear regression model with flexibility in the slope of the relationship between the forcing variable and outcomes of interest. We therefore parametrically estimate the following regression:

(1) $$Y_{im}=\alpha +\beta_{1}T_{m}+\beta_{2}G_{m}+\beta_{3}T_{m}G_{m}+\beta_{4}D_{m}+\beta_{5}G_{m}D_{m}+\varepsilon_{im}\;\forall |\mu_{m}|\leq h$$

where $T_{m}$ references the before and after periods ( $T_{m}=0$ before the program began, $T_{m}=1$ afterwards); $G_{m}$ references the CSR entry group; and $D_{m}$ represents the Euclidean distance to the cluster threshold. 13In the Appendix, we define the distance to the cluster threshold as $\mu (X_{ik})$.It is negative when closer to the 2006 entry group cluster center and positive when closer to the 2007 entry group cluster center. The indices i and m represent individuals and municipalities, respectively. The impact estimate is $\beta_{3}$, which measures the difference in intercepts at the threshold after the program begins. The coefficients $\beta_{4}$ and $\beta_{5}$ represent the local slopes with respect to the distance to the cluster threshold, which is allowed to vary by entry group. The bandwidth is represented by h, which varies in estimation.

### 4.4 Validity of RDD

**Figure 3:**
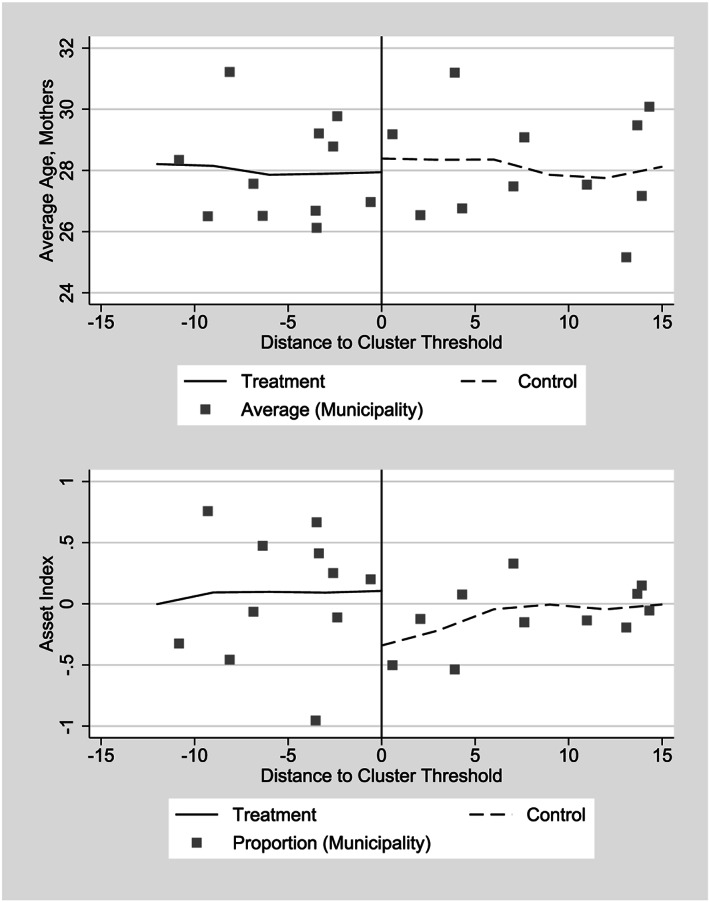
Difference in average values for selected control variables prior to entry to *Comunidades Solidarias Rurales*, 2006 and 2007 entry groups

For RDD estimates to be valid, observations just above and below the threshold must be similar in observable and unobservable characteristics. Though by definition the similarity of unobservable characteristics cannot be observed, we can compute and compare average values of observables on either side of the threshold (Table 1). Although we find no strictly statistically significant differences at the 5% level, the p value is less than .1 for one of six asset variables that we define (owning a sewing machine). For the two continuous variables with the lowest p values, the asset index, and the mother's age at time of childbirth, we graph them against the distance to cluster threshold prior to entry, to ensure that there is no obvious discontinuity at the threshold (Figure 3). In both cases, the municipal level averages (the points) appear relatively continuous at the threshold, so we can conclude the explanatory variables are balanced on either side of the implicit threshold.

**Table 1:** Comparing average values for covariates, between potential beneficiaries and non‐beneficiaries of *Comunidades Solidarias Rurales*(CSR), pre‐CSR entry

	CSR group	Comparison	p value
	mean	mean	
Age at time of childbirth (in years)	27.7	28.6	.240
	(0.4)	(0.6)	
Education: Third cycle (up to 9th year = 1)	0.188	0.156	.525
Education: Diploma or above (12th year and			
higher = 1)	0.078	0.090	.727
Marital status: Never married (=1)	0.104	0.156	.133
Marital status: Separated/divorced/widowed (=1)	0.075	0.114	.337
Log distance to health center (in km)	3.58	3.45	.508
	(0.13)	(0.16)	
Household assets			
Owns a radio/stereo (=1)	0.688	0.693	.890
Owns a fan (=1)	0.078	0.055	.574
Owns a sewing machine (=1)	0.052	0.086	.097
Owns a bicycle (=1)	0.251	0.171	.214
Owns a refrigerator (=1)	0.230	0.176	.292
Owns major furniture (other than beds) (=1)	0.199	0.162	.389
Household asset index	0.049	− 0.127	.313
	(0.149)	(0.082)	

*Note*. Standard errors for p values clustered at the municipality level; standard errors for means of continuous variables in parentheses. 389 total observations in 22 clusters. Asset index is the first principal component of the matrix defined by the six asset indicators listed. Source: CSR evaluation first and second survey.

**Figure 4:**
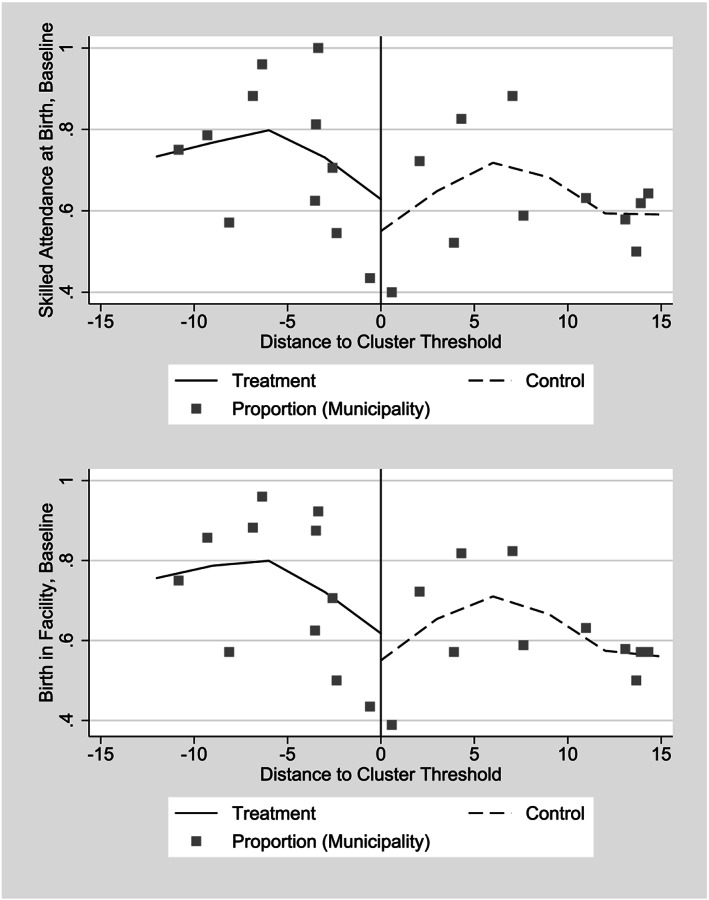
Average proportions, birth in facility and skilled attendance at birth and births in hospitals before *Comunidades Solidarias Rurales* began, 2006 and 2007 entry groups

Although we cannot ensure that outcomes would be continuous in absence of treatment, we can study their continuity related to the distance to cluster threshold before CSR began. First, we present average values among all mothers for all four outcome variables prior to the program (Table 2, Column 1). Reading the column downwards, we find no differences that exceed 3 percentage points between the 2006 and 2007 entry groups. To ensure that these averages do not mask changes near the threshold, we also graph these two variables against the distance to cluster threshold prior to CSR entry, and we find no initial differences (Figure 4). The figure strongly suggests both continuity at the threshold and a positive relationship between the variables and the distance to cluster threshold. 14Because the program was targeted at the municipal level, there are not enough degrees of freedom available to control for additional variables in the primary model. We relax distributional assumptions on standard errors in Tables B1 through B3 of Appendix S2 and condition on the explanatory variables used in Table 1, as well as the distance from the household to the health center. Results are not qualitatively different in these regressions from those described below.


**Table 2:** Descriptive statistics of maternal health service utilization indicators by entry group into *Comunidades Solidarias Rurales* (CSR)

	Preentry	Postentry	Difference	Difference‐in‐Difference	Sample size
Adequate prenatal care (5 or more visits)
2006 entry group (treatment group)	0.773	0.756	− 0.017	− 0.097	277
2007 entry group (control group)	0.744	0.824	0.080		233
Birth attended by skilled personnel
2006 entry group (treatment group)	0.659	0.903	0.244	0.217	254
2007 entry group (control group)	0.632	0.659	0.027		294
Gave birth in hospital
2006 entry group (treatment group)	0.633	0.903	0.270	0.180	254
2007 entry group (control group)	0.623	0.733	0.090		288
Postnatal checkup (first 2 weeks following birth)
2006 entry group (treatment group)	0.224	0.232	0.008	− 0.059	229
2007 entry group (control group)	0.192	0.259	0.067		258

*Source:* CSR evaluation first and second survey. Sample size varies slightly due to missing observations, particularly for postnatal checkups.

Following Imbens and Lemieux (2008), impact estimates primarily use local linear regression and a triangular kernel, reducing bias near the boundary (Fan & Gijbels, 1996; Hahn et al., 2001). 15We also show results with a rectangular kernel; all results are robust to using alternative kernels (e.g. Epanechnikov).All regressions have a relatively small number of clusters, raising the concern that statistical inference based on clustering will overreject the null hypothesis (e.g. Cameron et al., 2008). We therefore present p values based on wild cluster bootstrap t statistics, which are replicated 1,000 times for all estimates. 16See Imbens and Kalyanaraman (2012) and Bartalotti and Brummet (2017) for methods of choosing the bandwidth in sharp RDD applications.The p value represents the proportion of t ratios found in the acceptance region, and Cameron et al. (2008) demonstrate with a Monte Carlo that the wild cluster bootstrap does not overreject for as few as 10 clusters; all statements about statistical significance are based on these p values. We vary the bandwidth to test the sensitivity of results to inclusion or exclusion of observations farther away from the threshold. For all estimates, we use three different bandwidths (11, 8, and 5) to test whether coefficient magnitudes and significance change with bandwidth. 17We choose the most narrow bandwidth to ensure there are at least 10 communities in any regression, so statistical inference based on the wild bootstrap remains reliable.


## 5 RESULTS

We initially describe the proportion of mothers receiving adequate prenatal care, skilled attendance at birth, birth in a health facility, and postnatal care by entry group and by whether or not the care occurred pre‐ or posttreatment for the treatment group (Table 2). 18Straight difference‐in‐difference estimates for all dependent variables in the paper are also consistent with results described throughout the paper (Table B4 of Appendix S2).We find large increases in skilled attendance at birth and births in health facilities for the treatment group in the post‐2006 round, as well as modest increases in both indicators among the control group. For example, skilled attendance at birth increases from 65.9% for the treatment group in the preentry period to 90.3% in the postentry period, whereas among the control group it increases from 63.2 to 65.9%. However, there are no positive changes in prenatal or postnatal care among the treatment group, which average 75.6 and 23.2% of the sample, respectively, in the postentry period. In fact, in the control group, there are small increases in both indicators whereas changes are minimal among the treatment group.

**Figure 5:**
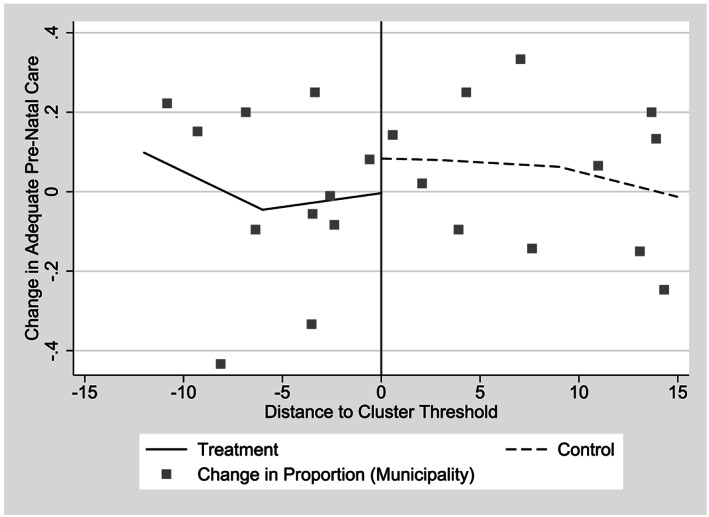
Change in proportion of births with adequate prenatal care, by distance from implied cluster threshold, 2006 and 2007 *Comunidades Solidarias Rurales* entry groups

The first outcome we examine is whether births took place after adequate prenatal care (Table 3). We find negative point estimates, but p values do not suggest that the differences from zero are statistically significant. The zero result is consistent with graphical evidence (Figure 5); when we examine municipal averages, we essentially observe no difference in the relationship between adequate prenatal monitoring and transfers associated with CSR. Although the program did not have a measurable impact on whether women received adequate prenatal monitoring, additional analysis shows that nearly all women did sign up for prenatal monitoring, and of those women who did not attend the minimum five visits considered adequate by the Salvadoran government, almost all attended four (de Brauw et al., 2010). In Table B1 of Appendix S2, we test alternative formulations of the dependent variable; specifically, the number of prenatal visits and an indicator for whether or not the first visit took place during the first 4 months of the pregnancy. In neither case is the treatment effect different from zero; therefore, we can conclude that CSR did not affect prenatal monitoring.

**Table 3:** Regression Discontinuity Design results for the impact of *Comunidades Solidarias Rurales* (CSR) on the proportion of births with adequate prenatal care, comparing 2006 entry to 2007 entry, using local linear regression

				Bandwidth		
		|Distance| < 11		|Distance| < 8		|Distance| < 5
Kernel	Rectangular	Triangular	Rectangular	Triangular	Rectangular	Triangular
Coefficient Estimate	− 0.124	− 0.128	− 0.126	− 0.121	− 0.118	− 0.102
	(0.076)	(0.078)	(0.092)	(0.077)	(0.101)	(0.073)
bootstrapped p value	.112	.130	.176	.144	.280	.206
Number of Obs.	441	441	367	367	270	270
Number of Clusters	18	18	14	14	10	10

Standard errors clustered by municipality in parentheses below coefficients. “ p value, unadjusted” is the p value associated with the standard error; the bootstrapped p value is based on the wild bootstrap procedure described by Cameron, Gelbach, and Miller (2008), and is not prone to false rejection of the null hypothesis with small numbers of clusters. Bandwidth refers to the Euclidean distance to the boundary between clusters, where observations (obs.) outside this distance are excluded from the sample. Source: CSR evaluation first and second survey.

This result might seem negative for the program implementation, as prenatal monitoring is one of the conditionality requirements of CSR. However, the conditionality was only binding among women who were pregnant at the time of the CSR census that preceded the first payment within a community. As a result, very few pregnancies were actually bound by the conditionality in practice; a mechanism to enroll newly pregnant women in the program could have increased the prevalence of prenatal monitoring. 19When the program was being implemented, program officials were quite concerned about inducing fertility (though no evidence shows CCTs induce fertility) through CSR payments so they did not want to provide benefits to newly pregnant mothers.


**Figure 6:**
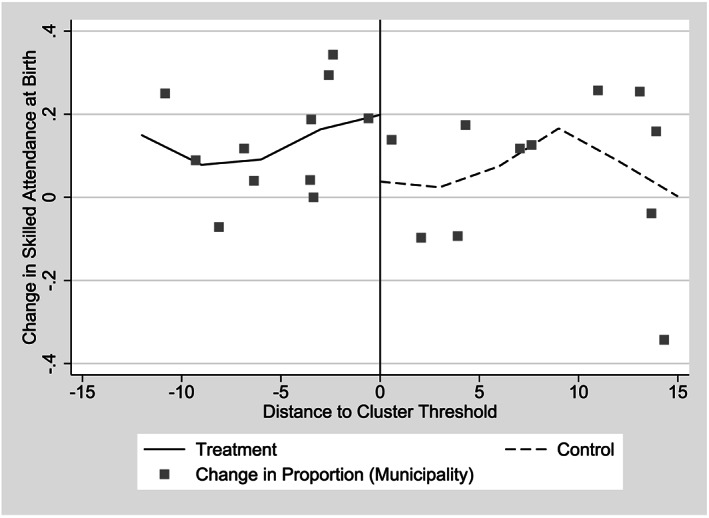
Change in the proportion of births with skilled attendance at birth by distance from implied cluster threshold, 2006 and 2007 *Comunidades Solidarias Rurales* entry groups

In contrast, we find strikingly positive results when examining the impact of CSR on skilled attendance at birth (Table 4). Point estimates of impacts range from 10.4 to 20.7 percentage points, with the smallest coefficient coming with the largest bandwidth and the rectangular kernel; some coefficients are significant at the 5 or 10% level, whereas the rectangular kernel coefficients have slightly higher p values. Graphically, the change in skilled attendance at birth is clear in municipal averages and appears to be about 20% close to the threshold (Figure 6), consistent with estimates from models estimated with preferred triangular kernels. Descriptive statistics show that the majority of this change is due to a shift away from attendance at birth by midwives (*parteras*) to attendance by obstetrician/gynecologists and other medical doctors. This finding is consistent with the government's initiative to “phase out” midwives, who were not recognized by the Ministry of Health's registration or training systems.

**Table 4:** Regression Discontinuity Design results for the impact of*Comunidades Solidarias Rurales* (CSR) on the proportion of births with skilled attendance at birth, comparing 2006 entry to 2007 entry, using local linear regression

				Bandwidth		
		|Distance| < 11		|Distance| < 8		|Distance| < 5
Kernel	Rectangular	Triangular	Rectangular	Triangular	Rectangular	Triangular
Coefficient estimate	0.104	0.164	0.148	0.180	0.207	0.171
	(0.063)	(0.064)	(0.059)	(0.072)	(0.092)	(0.085)
Bootstrapped p value	.128	.046	.028	.066	.112	.164
Number of Obs.	388	388	360	360	257	257
Number of Clusters	18	18	14	14	10	10

*Note*. Standard errors clustered by municipality in parentheses below coefficients. “ p value, unadjusted” is the p value associated with the standard error; the bootstrapped p value is based on the cluster bootstrap‐t procedure described by Cameron, Gelbach, and Miller (2008), and is not prone to false rejection of the null hypothesis with small numbers of clusters, the unadjusted p value is. Bandwidth refers to the Euclidean distance to the boundary between clusters, where observations (obs.) outside this distance are excluded from the sample. Control variables are listed in Table 1. Source: CSR evaluation first and second survey.

**Figure 7:**
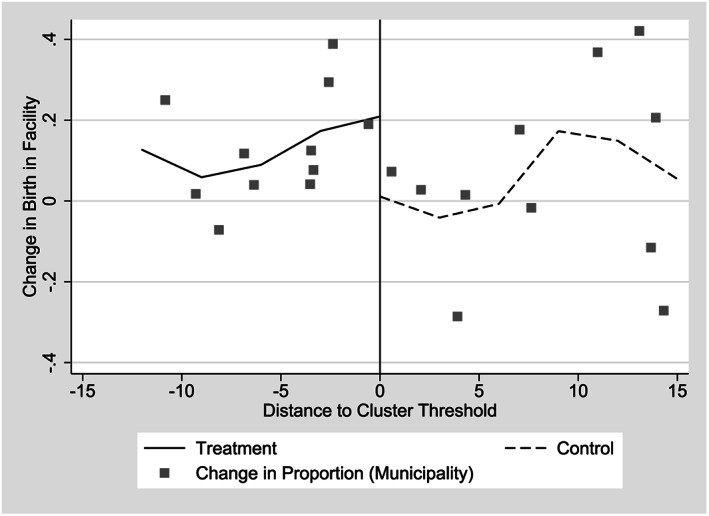
Change in the proportion of births in hospitals by distance from implied cluster threshold, 2006 and 2007 *Comunidades Solidarias Rurales* entry groups

We also find a large impact of CSR on a similar measure, births occurring in hospitals. Point estimates for impacts on births reported as taking place in hospitals largely mirror the results on skilled attendance at birth, though they are slightly larger and more likely to be statistically significant, ranging from 15.3 to 25.8 percentage points (Table 5). All estimates with the triangular kernel are significant at the 5% level or better. Graphically, we observe a steeper relationship between the proportion of births in hospitals at the municipal level and the forcing variable among the 2006 entry group than we observe in the same relationship for the 2007 entry group (Figure 7). Although there is a significant correlation between births taking place in hospitals and births attended by skilled professionals, the two measures do not fully overlap. However, both results suggest a significant program impact at ensuring mothers and infants had access to safe conditions to deal with complications at the time of birth.

**Table 5:** Regression discontinuity design results for the impact of*Comunidades Solidarias Rurales* (CSR) on the proportion of births in hospitals, comparing 2006 entry to 2007 entry, using local linear regression

				Bandwidth		
	|Distance| < 11		|Distance| < 8		|Distance| < 5	
Kernel	Rectangular	Triangular	Rectangular	Triangular	Rectangular	Triangular
Coefficient Estimate	0.153	0.211	0.199	0.223	0.258	0.204
	(0.070)	(0.059)	(0.066)	(0.061)	(0.081)	(0.067)
Bootstrapped p value	.060	.002	.010	.008	.020	.048
Number of Obs.	383	388	355	355	252	252
Number of Clusters	18	18	14	14	10	10

Notes: Standard errors clustered by municipality in parentheses below coefficients. “ p value, unadjusted” is the p value associated with the standard error; the bootstrapped p value is based on the cluster bootstrap‐t procedure described by Cameron, Gelbach, and Miller (2008), and is not prone to false rejection of the null hypothesis with small numbers of clusters, the unadjusted p value is. Bandwidth refers to the Euclidean distance to the boundary between clusters, where observations (obs.) outside this distance are excluded from the sample. Control variables are listed in Table 1. Source: CSR evaluation first and second survey

**Figure 8:**
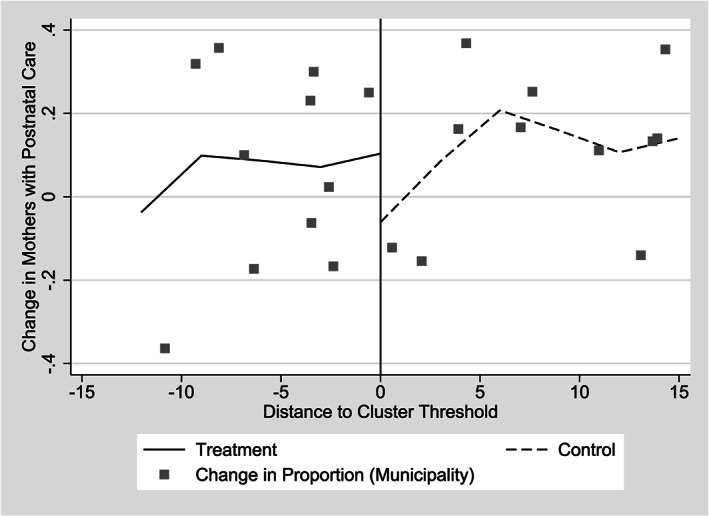
Change in the proportion of births with postnatal care by distance from implied cluster threshold, 2006 and 2007 *Comunidades Solidarias Rurales* entry groups

We next estimate the impact of CSR on whether or not women obtain postnatal care in the 2 weeks after birth (Table 6). We largely find negative point estimates that are not significantly different from zero. Graphical evidence also indicates no difference at the threshold, again suggesting no program impact (Figure 8). In Table B3 of Appendix S2, we use an alternative indicator, whether or not women received postnatal care within first 6 weeks after birth and again find no statistically significant program impact. Clearly, if messages about the importance of receiving any postnatal care are part of CSR, they are either not being received by women, or being confused with messaging surrounding other health care, such as infant growth monitoring check‐ups.

**Table 6:** Regression Discontinuity Design results for the impact of*Comunidades Solidarias Rurales* (CSR) on the proportion of births with postnatal care in first 2 weeks, comparing 2006 entry to 2007 entry, estimated with local linear regression

			Bandwidth			
	|Distance| < 11		|Distance| < 8		|Distance| < 5
Kernel	Rectangular	Triangular	Rectangular	Triangular	Rectangular	Triangular
Coefficient Estimate	‐0.117	− 0.152	− 0.174	− 0.166	− 0.196	− 0.160
	(0.120)	(0.125)	(0.117)	(0.139)	(0.177)	(0.146)
Bootstrapped p value	0.336	0.296	0.204	0.248	0.298	0.418
Number of Obs.	343	343	316	316	221	221
Number of Clusters	16	16	14	14	10	10

*Note*. Standard errors clustered by municipality in parentheses below coefficients. “ p value, unadjusted” is the p value associated with the standard error; the bootstrapped p value is based on the cluster bootstrap‐t procedure described by Cameron, Gelbach, and Miller (2008), and is not prone to false rejection of the null hypothesis with small numbers of clusters, the unadjusted p value is. Bandwidth refers to the Euclidean distance to the boundary between clusters, where observations (obs.) outside this distance are excluded from the sample. Control variables are listed in Table 1. Source: CSR evaluation first and second survey.

In sum, we find impacts on outcomes at birth, including strong evidence on whether or not the birth took place within a hospital, and slightly weaker evidence on whether or not a skilled attendant was present for the birth. However, we find neither impacts of program impacts on prenatal nor postnatal care for mothers. In Section 6, we discuss possible mechanisms.

## 6 DISCUSSION AND CONCLUSION

Previous research on cash transfers and maternal health outcomes has largely failed to identify potential impact pathways. Based on the hypothesized six impact pathways discussed earlier, we argue two potential pathways are likely to have contributed to the impacts of CSR, in addition to the income effect: supply‐side health service improvements and gains in women's decision‐making agency. 20Although we cannot directly isolate the income effect, the size of the average transfer was less than 10% of household monthly income, so we hypothesize this effect is likely to be small, if it exists.Before discussing these two pathways in detail, we first explain why other pathways are less likely to be responsible for impacts we observe. First, as we did not find impacts on the only health care utilization variable with explicit conditions (prenatal care), we can dismiss the role of conditionalities as a primary driver of impacts. To completely rule out the role of conditions, we would need to experimentally design a further study. However, in this context, if participants understood the program rules as binding, the role of conditions appear to be limited. The possibility of increased health information is a potential pathway in CSR, as health training sessions were an integral part of service delivery and offered monthly at a local meeting point, such as a church, school, or government building. Although not officially required for program recipients, 75% of beneficiaries attended health training sessions in the month previous to the second survey, and program recipients largely believe attendance is required for payments. However, the data do not suggest the early entry group had higher exposure to training modules on either infant and child health or family health. In fact, reports of ever having attended training on either subject are higher among the later entry group than among the early entry group (66.6 versus 52.8% for infant and child health; 45.1 vs. 38.4% for family health). Therefore, although health training sessions may contribute to increases in health‐seeking behavior, they cannot be demonstrably linked to program impacts on the measures we examine.

In contrast, a plausible pathway responsible for CSR's increase in demand for maternal health care is through gains in women's decision‐making agency and empowerment. Qualitative and ethnographic evidence collected through the IFPRI–FUSADES evaluation (Adato et al., 2009) find CSR increased women's decision‐making agency by increasing the share of income controlled by women and through participation in health‐training sessions, in which women in the community interacted and built social networks. Qualitative and quantitative evidence indicates that women often traveled together to meeting places for both health training sessions and transfer receipt, so information about new options for giving birth may have spread through conversations either at health training sessions or in transit. Measures of women's empowerment are difficult to quantify in impact evaluations, and our data lack any such measures to be able to test this mechanism formally (Peterman et al., 2015). However, the qualitative results imply this pathway is important for maternal health outcomes in El Salvador, especially in combination with the increased availability of health care discussed below.

Second, basic infrastructure, service coverage, and quality of health services improved during early years of CSR implementation. Based on findings from other programs, supply‐side investments such as expanding provider coverage and quality of care are essential and important components for program success (e.g. Powell‐Jackson & Hanson, 2012). Health facility surveys were undertaken during the first and third survey rounds; thus, although the measurements are not strictly comparable to the time frame for this analysis, they demonstrate a trend of service improvements. We find there were improvements in both availability and quality of health services; however, again, trends in communities among the early entry group are not significantly higher than those in the later entry group (de Brauw et al., 2010). We do find a larger number of skilled personnel (doctors of any kind and trained nurses) being present in early treatment communities postentry, suggesting potential preferential results for outcomes at birth, but we are not able isolate this impact. Furthermore, we lack information on the timing of supply‐side improvements, so we have to assume they largely took place at the same time in the 2006 and 2007 entry groups. Therefore, we conclude that if health infrastructure improvements contributed to impacts, it was likely in combination with demand increases.

The results are limited by the lack of more specialized indicators of prenatal or postnatal care quality, or infant/maternal mortality, which would have allowed for more nuanced findings. Indeed, recent evidence has emphasized the content and quality of maternal health care, rather than the absolute number or timing of visits as key to preventing mortality and morbidity of mother and child (Miller et al., 2016). Because of the rapid program expansion, the time frame over which we analyze changes is short, and it is possible that with a different evaluation design, we could have had more opportunity to observe changes. Despite these limitations, results imply that there are important nuances to program design and implementation that should be taken into account for a poverty‐focused CCT to successfully affect maternal health outcomes. For example, the results suggest conditions may not be a primary driver of potential impacts on health service utilization, possibly because women who are able are already meeting the required thresholds for conditions. If so, unconditional transfers, as found in many national programs in Africa, may work via similar pathways as found here. Consequently, if a program objective is maternal health, a more effective program design would be to require contact with health facilities in the first 3 months of pregnancy as part of program enrollment (rather than conditioning and monitoring each recommended visit), followed by behavioral nudges and messaging around the importance of skilled birth attendance and postnatal care within 2 weeks of birth. The latter may be especially important in settings like El Salvador, where the rates of postnatal care are quite low. Further, program designs with linkages to health insurance waivers or subsidies in settings where maternal health care is not free is also promising. Interactions with health services across a more sustained period also have a greater opportunity to address a variety of maternal health concerns. For example, as part of postnatal care, family‐planning counseling and cervical cancer screening could be required, both of which are important components that may be omitted during prenatal care, when the focus is on the pending pregnancy and birth planning.

As more cash transfer programs are scaled‐up, future impact evaluations could more rigorously examine maternal health outcomes, particularly in Africa and Asia. Recent rigorous evaluations of diverse schemes in these settings have shown promise in some cases, although questions remain as to the role of program design and impact pathways (Handa et al., 2016; Triyana & Shankar, 2017; Cohen et al., 2017). The impacts and cost effectiveness of poverty and human capital focused social protection schemes should be carefully considered, alongside targeted programs such as health vouchers and removal of user fees. Because of sample size limitations, attention to these components may require oversampling pregnant mothers in the baseline data or collecting more detailed information on fertility, prenatal care, and birth indicators, going beyond utilization as used in the current study. Although the range of potential indicators is diverse, potential measures include quality of care (e.g. service coverage of recommended services and care during pregnancy and postpartum) and health outcomes (e.g. low birth weight and morbidity rates) matched with supply‐side measures of health infrastructure (Moller et al., 2018). Methodologically, if RDD methods are to be used in evaluating impacts, we suggest oversampling near the threshold for additional power in estimation. The lack of rigorous evidence on maternal health service utilization, as well as health outcomes, is a limiting factor in advancing and making sound recommendations on design and implementation of CCTs, voucher schemes, and other financial incentives (Morgan et al., 2012). In light of the current public health and nutrition emphasis on the first 2 years of life as critical windows of opportunity for determining future health, education, and labor‐force outcomes, failing to include attention to maternal health is a missed opportunity. The views expressed in this article are those of the authors and not the policies or views of their affiliated institutions; neither authors have a conflict of interest. We thank Margarita Beneke de Sanfeliu, Mauricio Arturo Shi Artiga, and others at the *Fundación Salvadoreña para el Desarrollo Económico y Social* (FUSADES) for helpful conversations and excellent data collection, to Daniel Gilligan for contributions to methodology, and to the editor and two anonymous reviewers for helpful comments. We would also like to thank participants in the 2011 Population Association of America annual meetings in Washington DC and the June, 2011 3ie conference in Cuernavaca, México, for helpful comments, and Mauricio Sandoval of the *Fondo de Inversión Social para el Desarrollo Local* (FISDL) for guidance throughout the evaluation process. The evaluation data used in the study were collected on behalf of the Government of El Salvador by funding through FISDL. All errors are our own.

## Supporting information

Appendix SSM.docx
