# Supplementary material for: Can conditional cash transfers improve maternal health care? Evidence from El Salvador's Comunidades Solidarias Rurales program
Source: Health Econ. 2020 Mar 2;29(6):700–15. doi: 10.1002/hec.4012 (PMC7383823; doi:10.1002/hec.4012)
Supplement: Supplementary file 1 — Appendix SSM.docx [file HEC-29-700-s001.docx]

**Appendix A. Partitioned Cluster Analysis and RDD**

Partitioned cluster analysis is a set of iterative methods of splitting observations in data sets into distinct groups similar across multiple characteristics. Observations are grouped along those attributes using an iterative procedure as follows. First, the analyst chooses the number of groups, *K*, that will be formed. Second, the analyst chooses *K* initial cluster centers, calculates the distance from each observation to each initial cluster center, and assigns each observation to the cluster with the nearest center. The centers of each cluster are recalculated as either the mean or median of the points aligned with that center. The process is then repeated; the distances to the new *K* cluster centers are calculated for each observation, and each observation is assigned to the nearest cluster center. If any observations have switched clusters, then new cluster centers are again computed. This procedure is repeated until no observations switch clusters.

To summarize, initial choices are made about the number of clusters, cluster centers, the distance metric to use, and the method of choosing new cluster centers as iteration occurs (mean or median). Conditional on those choices, results of partitioned cluster analysis will always be the same. Mathematically, if we define the center of the *k*-th cluster as, and the distance between any observation *i* with a vector of traits $\boldsymbol{X}_{\boldsymbol{i}}$ and $\eta_{k}$ can be defined as $d({\boldsymbol{X}_{\boldsymbol{i}},\eta}_{k})$. Cluster membership is defined as the cluster *k* for which the distance to the cluster center is smallest. We therefore define a vector of all $\boldsymbol{X}_{\boldsymbol{i}\boldsymbol{k}}$ such that $\boldsymbol{X}_{\boldsymbol{ik}}=\underset{k\epsilon K}{\mathrm{argmax}} d({\boldsymbol{X}_{\boldsymbol{i}},\eta}_{k})$. We use the high poverty group, entering CSR in 2006, as the treatment group, and the moderate poverty group, entering in 2007, as the control group.^[[1]](#footnote-1)^ The threshold between the two groups can be defined as all points equidistant from $\eta_{h}$ and $\eta_{m}$, the final cluster centers, where *h* and *m* index high and moderate poverty, respectively. To target CSR, the Euclidean distance to the cluster centers was used in the clustering procedure. The equidistant line from the cluster centers can be defined by the equation $d\left( {\boldsymbol{W}_{\boldsymbol{j}},\eta}_{h} \right)=d\left( {\boldsymbol{W}_{\boldsymbol{j}},\eta}_{m} \right)$, where we call $\boldsymbol{W}_{\boldsymbol{j}}$ the set of points making up the cluster threshold. Then the distance to the cluster threshold is defined as the distance, µ, between a municipality's traits $\boldsymbol{X}_{\boldsymbol{ik}}$ and its assigned cluster center $\boldsymbol{W}_{\boldsymbol{j}}$, which is written mathematically as:

${\mu(\boldsymbol{X}}_{ik})=\left| \boldsymbol{X}_{\boldsymbol{ik}}-\boldsymbol{W}_{\boldsymbol{j}} \right|$ (A.1)

Points that are arbitrarily close to the cluster threshold would be points for which a small change in one of the indicators making up $\boldsymbol{X}_{\boldsymbol{ik}}$, would cause a switch from a treatment to a control cluster, or vice-versa. Therefore, the distance to cluster threshold µ acts as the forcing variable in RDD.^[[2]](#footnote-2)^ In parallel with a more explicit forcing variable, small shifts in the values of specific components of the cluster analysis can shift observations close to the threshold from one cluster to another, whereas observations farther from the cluster threshold would be less similar.^[[3]](#footnote-3)^ Our primary estimation strategy, then, is to use the distance to cluster threshold as the forcing variable and sharp RDD to estimate impacts.

To use $\mu\left( \cdot\right)$ as the forcing variable, two of the three main assumptions critical for the consistency of the sharp RDD estimator must be examined.^[[4]](#footnote-4)^ It is necessary to assume that observations just on either side of the threshold are similar in both observed and unobserved characteristics, and that any outcome $Y_{i}$ would be continuous at the threshold in the absence of a program. If the assumptions needed to define the implicit distance metric and these two assumptions hold, the distance to cluster threshold is valid as an RDD forcing variable to generate consistent program impacts.

**Appendix B. Additional Control Variables**

Although methodologically it is not necessary to include other control variables in our estimation equations to identify the impact of CSR, we include several control variables in alternative versions of our estimates to both ensure that they are relatively balanced on either side of the threshold. We include them in some versions of the estimation to ensure that their inclusion does not affect parameter estimates. In theory, they should explain some additional variance in the outcome, though they use scarce degrees of freedom.

We include variables at the individual, household, and community level. The individual-level control variables included are the mother’s age in years, indicators for achieving specific levels of educational attainment, and marital status (married or not), as well as divorced or separated. The education indicators include completion of the third cycle (years 7-9) and completion of the diploma or above (years 12 and higher), while primary (up to year 6) or no schooling are omitted. At the household level, we include an index of household asset holdings created using principal components analysis (e.g. Filmer and Pritchett 2001). Assets included in the factor score are as listed in Table 1. Note that this variable could have been affected by the program, as households have additional cash flow from the program, and the 2006 entry group would have had such cash flow for a longer period of time. Finally, at the community level the logarithm of distance to the nearest health center (in kilometers) is included to control for access to health services.

***Appendix Tables***

**Appendix Table B.1. Regression Discontinuity Design results for the impact of *Comunidades Solidarias Rurales* on measures of adequate prenatal care, comparing 2006 entry to 2007 entry, controlling for individual and household characteristics**

| Dependent Variable | Adequate prenatal care (5 or more visits) | | | Number of prenatal care visits | | | First visit took place within first 4 months of pregnancy | |
| --- | --- | --- | --- | --- | --- | --- | --- | --- |
|  | **(1)** | **(2)** | **(3)** | | **(4)** | **(5)** | | **(6)** |
| Time Period | 0.054 | 0.051 | 0.104 | | 0.075 | 0.010 | | 0.013 |
|  | (0.055) | (0.056) | (0.320) | | (0.320) | (0.060) | | (0.059) |
| Treatment Group | -0.075 | -0.057 | -0.417 | | -0.268 | 0.112 | | 0.128 |
|  | (0.074) | (0.076) | (0.446) | | (0.450) | (0.073) | | (0.074) |
| CSR (Time Period * Treatment Group) | -0.067 | -0.050 | 0.074 | | 0.114 | -0.118 | | -0.099 |
|  | (0.076) | (0.076) | (0.465) | | (0.461) | (0.079) | | (0.078) |
| Distance to Cluster Threshold | -0.002 | 0.002 | 0.020 | | 0.050 | -0.015 | | -0.011 |
| * Treatment Group | (0.010) | (0.010) | (0.063) | | (0.064) | (0.009) | | (0.009) |
| Distance to Cluster Threshold | -0.004 | -0.004 | -0.043 | | -0.042 | 0.009 | | 0.010* |
|  | (0.005) | (0.005) | (0.031) | | (0.031) | (0.005) | | (0.005) |
| Mother's Age (in years) |  | -0.001 |  | | -0.009 |  | | 0.002 |
|  |  | (0.003) |  | | (0.018) |  | | (0.003) |
| Third Cycle School (9th year; 1=yes) |  | 0.091 |  | | 0.416 |  | | 0.111** |
|  |  | (0.051)* |  | | (0.287) |  | | (0.045) |
| Diploma or above (12 years; 1=yes) |  | 0.077 |  | | 0.780 |  | | 0.040 |
|  |  | (0.062) |  | | (0.553) |  | | (0.065) |
| Not married (1=yes) |  | -0.085 |  | | -0.682 |  | | -0.090 |
|  |  | (0.066) |  | | (0.353) |  | | (0.066) |
| Divorced, separated or widowed (1=yes) |  | 0.009 |  | | -0.371 |  | | 0.025 |
|  |  | (0.067) |  | | (0.397) |  | | (0.063) |
| Asset Index (continuous) |  | -0.005 |  | | 0.032 |  | | <-0.001 |
|  |  | (0.015) |  | | (0.079) |  | | (0.014) |
| Log distance to health center (km) |  | 0.016 |  | | 0.234** |  | | 0.025 |
|  |  | (0.021) |  | | (0.108) |  | | (0.020) |
|  |  |  |  | |  |  | |  |
| Number of observations | 510 | 504 | 510 | | 504 | 486 | | 481 |
| R^2^ | 0.006 | 0.018 | 0.004 | | 0.030 | 0.016 | | 0.032 |

Notes: Standard errors clustered at the municipality level in parentheses. Regressions correspond to results controlling for individual, household, and community characteristics in Table 3. Services index is a sum of indicators for piped water, electricity, and the presence of a toilet. *-indicates significance at the 10 percent level; **- indicates significance at the 5 percent level; ***- indicates significance at the 1 percent level.

**Appendix Table B.2. Regression Discontinuity Design results for the impact of *Comunidades Solidarias Rurales* on skilled attendance and birth in facility, comparing 2006 entry to 2007 entry, controlling for individual and household characteristics**

| Dependent Variable | Skilled Attendance at Birth | | Birth in Facility | |
| --- | --- | --- | --- | --- |
|  | **(1)** | **(2)** | **(3)** | **(4)** |
| Time Period | 0.024 | 0.034 | 0.011 | 0.013 |
|  | (0.060) | (0.060) | (0.061) | (0.061) |
| Treatment Group | -0.029 | -0.044 | -0.031 | -0.057 |
|  | (0.082) | (0.081) | (0.083) | (0.082) |
| CSR (Time Period * Treatment Group) | 0.151* | 0.141* | 0.175** | 0.162* |
|  | (0.078) | (0.078) | (0.078) | (0.079) |
| Distance to Cluster Threshold | -0.022* | -0.021* | -0.023** | -0.022** |
| * Treatment Group | (0.011) | (0.010) | (0.011) | (0.010) |
| Distance to Cluster Threshold | -0.001 | -0.003 | -0.002 | -0.004 |
|  | (0.006) | (0.006) | (0.006) | (0.006) |
| Mother's Age (in years) |  | 0.002 |  | <0.001 |
|  |  | (0.003) |  | (0.003) |
| Third Cycle School (9th year; 1=yes) |  | 0.106* |  | 0.112** |
|  |  | (0.052) |  | (0.052) |
| Diploma or above (12 years; 1=yes) |  | 0.143** |  | 0.121* |
|  |  | (0.061) |  | (0.063) |
| Not married (1=yes) |  | 0.095 |  | 0.077 |
|  |  | (0.058) |  | (0.059) |
| Divorced, separated or widowed (1=yes) |  | 0.109 |  | 0.086 |
|  |  | (0.062) |  | (0.065) |
| Asset Index (continuous) |  | 0.018 |  | 0.028* |
|  |  | (0.015) |  | (0.015) |
| Log distance to health center (km) |  | -0.050** |  | -0.048** |
|  |  | (0.018) |  | (0.018) |
|  |  |  |  |  |
| Number of observations | 551 | 545 | 545 | 539 |
| R^2^ | 0.046 | 0.085 | 0.053 | 0.093 |

Notes: Standard errors clustered at the municipality level in parentheses. *-indicates significance at the 10 percent level; **- indicates significance at the 5 percent level.

**Appendix Table B.3. Regression Discontinuity Design results for the impact of *Comunidades Solidarias Rurales* on measures of postnatal care, comparing 2006 entry to 2007 entry, controlling for individual and household characteristics**

| Dependent Variable | Postnatal care within first two weeks | | Postnatal care within first six weeks | |
| --- | --- | --- | --- | --- |
|  | **(1)** | **(2)** | **(3)** | **(4)** |
| Time Period | 0.039 | 0.039 | 0.073 | 0.094 |
|  | (0.053) | (0.055) | (0.066) | (0.067) |
| Treatment Group | 0.070 | 0.066 | 0.026 | 0.020 |
|  | (0.080) | (0.081) | (0.092) | (0.093) |
| CSR (Time Period * Treatment Group) | -0.054 | -0.069 | -0.011 | -0.038 |
|  | (0.086) | (0.086) | (0.101) | (0.103) |
| Distance to Cluster Threshold | 0.023** | 0.024** | 0.013 | 0.014 |
| * Treatment Group | (0.010) | (0.010) | (0.012) | (0.012) |
| Distance to Cluster Threshold | -0.009* | -0.009* | -0.008 | -0.009 |
|  | (0.005) | (0.005) | (0.006) | (0.007) |
| Mother's Age (in years) |  | -0.001 |  | 0.002 |
|  |  | (0.003) |  | (0.003) |
| Third Cycle School (9th year; 1=yes) |  | -0.035 |  | -0.047 |
|  |  | (0.053) |  | (0.066) |
| Diploma or above (12 years; 1=yes) |  | 0.099 |  | 0.106 |
|  |  | (0.079) |  | (0.082) |
| Not married (1=yes) |  | -0.054 |  | 0.017 |
|  |  | (0.055) |  | (0.072) |
| Divorced, separated or widowed (1=yes) |  | 0.011 |  | 0.043 |
|  |  | (0.074) |  | (0.086) |
| Asset Index (continuous) |  | 0.013 |  | 0.011 |
|  |  | (0.016) |  | (0.018) |
| Log distance to health center (km) |  | 0.003 |  | 0.026 |
|  |  | (0.018) |  | (0.022) |
|  |  |  |  |  |
| Number of observations | 489 | 484 | 489 | 484 |
| R^2^ | 0.016 | 0.025 | 0.010 | 0.022 |

Notes: Standard errors clustered at the municipality level in parentheses. *-indicates significance at the 10 percent level; **- indicates significance at the 5 percent level.

**Appendix Table B.4. Difference-in-Difference estimates, all outcome variables**

| Outcome Variable | No Additional Controls | Controlling for Distance to Cluster Threshold | Controlling for Distance to Cluster Threshold and Asset Index |
| --- | --- | --- | --- |
| Adequate prenatal care | -0.063  (0.073) | -0.067  (0.073) | -0.060  (0.072) |
| Number of prenatal visits | 0.111  (0.453) | 0.080  (0.439) | 0.144  (0.446) |
| Any prenatal visit in first 4 months | -0.124  (0.090) | -0.122  (0.090) | -0.112  (0.089) |
| Skilled attendance at birth | 0.147**  (0.068) | 0.146**  (0.065) | 0.145**  (0.064) |
| Birth in facility | 0.162*  (0.080) | 0.162**  (0.077) | 0.161**  (0.074) |
| Postnatal visit within 2 weeks | -0.058  (0.098) | -0.057  (0.098) | -0.057  (0.099) |
| Postnatal visit within 6 weeks | -0.017  (0.082) | -0.017  (0.082) | -0.017  (0.081) |

Notes: Each cell represents a separate estimation. Standard errors clustered at the municipality level in parentheses. *-indicates significance at the 10 percent level; **- indicates significance at the 5 percent level.

1. Values for the poverty rate and the severe stunting rate for each municipality in the 2006 and 2007 entry groups are shown in Figure 2 of the main paper. [↑](#footnote-ref-1)
2. As defined, the distance to cluster threshold is quite similar to the use of the spatial distance to a city or geographic boundary as a forcing variable (e.g. Lavy, 2010). [↑](#footnote-ref-2)
3. Note that in the case of CSR, the line is almost vertical, and using the poverty rate as a forcing variable does not change the assignment of municipalities to groups; hence, it can be used as an alternative forcing variable. All results in the paper are robust to using the poverty rate alone as an alternative forcing variable. [↑](#footnote-ref-3)
4. The first assumption, that the probability of treatment varies discontinuously at the threshold, clearly applies. [↑](#footnote-ref-4)
